# The Influence of Posture on Heart Murmurs

**Published:** 1902-03-22

**Authors:** 


### THE INFLUENCE OF POSTURE ON HEART MURMURS.

Dr. W. Gordon[1] has carefully studied the influence of posture on cardiac bruits, and shows that the subject deserves far more attention than has hitherto been bestowed upon it; the effects produced upon the cardiac sounds by a change of position from the sitting to the recumbent position being sometimes profound. He states as a preliminary that there are certain errors which must be avoided. The influence of exertion must be eliminated by taking care that the change of position is effected by aid of an assistant, so that no effort is made by the patient himself; the transitory effect of the changed condition must be allowed to pass away by letting a few heart-beats go by before listening again to the heart, and the possible influence of altered adjustment of the stethoscope must be got rid of by making all the observations with a straight wooden stethoscope. Moreover all cases of "cardio-pulmonary" murmurs should be excluded. It is to be noted in regard to the diagnosis of these latter murmurs that Dr. Gordon entirely dissents from Potain's sweeping statement that all murmurs which almost or altogether disappear when a change is made from the sitting to the recumbent position or *vice versâ* are of a cardio-pulmonary character, holding that this statement is not in accordance with the facts. It is evident, however, that by insisting on this point Dr. Gordon draws into his net a certain number of murmurs which would be excluded as "cardio-pulmonary" murmurs by other observers.

After describing fully the changes in the intensity of various bruits caused by change of posture, Dr. Gordon enters into a consideration of the causes of these alterations and comes to the conclusion that they are largely due to change in chest-depth and to the varying influence of gravity in the different positions of the body. He shows that although a rigid chest may not differ at all in the two positions (upright and recumbent), a very elastic chest may be as much as one inch deeper antero-posteriorly in the upright position. The average difference between the antero-posterior diameters of the upright and recumbent chest at the nipple level seems to be half to three-quarters of an inch which, deducting the large space occupied by the solid structures of the back, means that the space which contains the heart becomes, in such a patient, on recumbency, shallower by about one-sixth of its depth, thus bringing the listening ear appreciably closer to the murmur-producing orifice. The varying effect of gravity upon the blood stream according as the patient is in the upright or the recumbent position is sufficiently obvious, and Dr. Gordon summarises the results of his observations by saying, "It seems to me (1) that recumbency tends to increase all 'hæmic murmurs' except the venous hum, which it tends to obliterate; to increase the murmurs of mitral regurgitation, tricuspid regurgitation, and aortic stenosis; to decrease the murmur of mitral stenosis; and to leave little, if at all, affected the murmur of aortic regurgitation. (2) That the effects of gravity and change in chest-depth seem to account for the influence of recumbency. And (3) That, therefore, in describing and discussing murmurs which posture modifies, the patient's position at the time of observation should be stated."

[1] Brit. Med. Jour., March 15.

